# The Role of Oxygen Sensors, Hydroxylases, and HIF in Cardiac Function and Disease

**DOI:** 10.1155/2015/676893

**Published:** 2015-09-28

**Authors:** W. H. Davin Townley-Tilson, Xinchun Pi, Liang Xie

**Affiliations:** Department of Medicine, Cardiovascular Research Institute, Baylor College of Medicine, Houston, TX 77030, USA

## Abstract

Ischemic heart disease is the leading cause of death worldwide. Oxygen-sensing proteins are critical components of the physiological response to hypoxia and reperfusion injury, but the role of oxygen and oxygen-mediated effects is complex in that they can be cardioprotective or deleterious to the cardiac tissue. Over 200 oxygen-sensing proteins mediate the effects of oxygen tension and use oxygen as a substrate for posttranslational modification of other proteins. Hydroxylases are an essential component of these oxygen-sensing proteins. While a major role of hydroxylases is regulating the transcription factor HIF, we investigate the increasing scope of hydroxylase substrates. This review discusses the importance of oxygen-mediated effects in the heart as well as how the field of oxygen-sensing proteins is expanding, providing a more complete picture into how these enzymes play a multifaceted role in cardiac function and disease. We also review how oxygen-sensing proteins and hydroxylase function could prove to be invaluable in drug design and therapeutic targets for heart disease.

## 1. Introduction

Ischemic heart disease is the leading cause of death in the world, killing an estimated 7.4 million people annually [1]. Currently, in the US alone, more than 5 million people live with heart failure, primarily due to ischemia [2, 3]. Ischemia starves the heart of vital oxygen, leading to the death of cardiomyocytes. Because cardiomyocytes are generally thought of as postmitotic [4, 5], any cell death leads to a permanent reduction of heart function. Whether gradual, as seen in atherosclerotic plaque buildup, or acute, as in myocardial infarction, this oxygen-dependent damage to the heart is responsible for the vast majority of cardiac-related mortality. Therefore, the role of oxygen in cardiac function is essential in understanding how to ameliorate the effects of heart disease.

Oxygen is to be a central mediator of myriad protein functions and biochemical reactions. One of oxygen's primary roles is producing adenosine triphosphate (ATP) through oxidative phosphorylation during aerobic cellular respiration [6], during which nicotinamide adenine dinucleotide (NADH) and flavin adenine dinucleotide (FADH_2_) donate their electrons to oxygen, producing potential energy. This energy is then utilized by ATP synthase to generate ATP from ADP via phosphorylation. While this process is amazingly efficient in its ability to generate energy for the cell, converting up to 36 ATP molecules per one molecule of glucose [7], it also has the potential to generate nitric oxide (NO) and reactive oxygen species (ROS) like superoxide anion (O_2_^−^) and hydrogen peroxide (H_2_O_2_) [8]. These products have the potential to influence many disease states through signaling pathways and the damaging effects of free radicals on cell health. Thus, it is important to note that while oxygen plays a very beneficial role in cell metabolism and global organismal health, its byproducts can also mediate detrimental consequences in heart health including cardiomyocyte hypertrophy, excitation-contraction coupling, arrhythmia, and cell viability [9].

Though still under active study, the role of oxygen as a substrate for enzymatic function has been widely accepted as yet another regulator of many cellular processes. Currently, there are over 200 unique enzymes that use oxygen as a substrate [10] (Tables 1 and 2): these enzymes are typically broken down into two major categories, oxidases and oxygenases. Oxidases typically use a metal or flavin coenzyme to catalyze the oxidation of a substrate without incorporating oxygen into the main product, instead using oxygen as the electron acceptor [11]. In contrast, oxygenases incorporate oxygen molecules into the substrate as a hydroxyl or carboxyl group in a two-step process, (1) the binding of the oxygen molecule to the catalytic domain of the enzyme and (2) the subsequent transference of the oxygen to the substrate [12].

As part of the oxygenase supergroup, hydroxylases are known to be increasingly important in the context of mediating numerous cellular processes. Collagen is one of the most well-known proteins whose function is mediated by hydroxylation state, whereby both proline and lysine hydroxylation enhance the “tightness” of the triple alpha-helix structure [13, 14]. Other notable hydroxylated proteins are the hypoxia inducible factors (HIFs), which are described in greater detail below in the section “Hydroxylases and Hydroxylation in the Heart”. HIFs play a major role during hypoxia in cell proliferation [15], angiogenesis [16], and embryonic vasculogenesis [17]. Interestingly, HIF-1*α* signaling is dictated by hydroxylation in two ways: via von Hippel-Lindau- (VHL-) mediated degradation in the proteasome if prolines 402/564 (in humans) are hydroxylated by prolyl hydroxylase domain protein (PHD) [18–20] or via transactivational inhibition if asparagine 803 residue is hydroxylated by the factor inhibiting HIF (FIH) [21–23]. The inactivation of HIF, either through degradation or transactivational inhibition, directly impacts over 100 genes with functional consequences such as angiogenesis, metabolic adaptation, metastasis, apoptosis, and more [24–27] and indirectly impacts even more, as is the case for the p53 pathway [28] endothelial transcription factors [29] and genes regulated by histone methylation [30]. Interestingly, HIF activity can also be enhanced through proline hydroxylation of its regulator pyruvate kinase M2 (PKM2), which increases the interaction between HIF and PKM2, increasing HIF transactivation and transcriptional activity [31]. While HIF signaling dominates the literature in terms of what is known about hydroxylation signaling in the heart, other cardiac events mediated by hydroxylation are becoming more appreciated. Of note, the *β*-adrenergic receptors (*β*-ARs), similar to HIF, can be hydroxylated by PHD proteins and subsequently targeted for ubiquitin-mediated degradation [32]. Other cardiovascular targets of hydroxylation include inhibitor of *κ*B kinase *β* (IKK*β*) [33] and myogenin [34], though the role of these two proteins is unclear in regard to hypoxic stimuli. As the list of hydroxylated cardiac proteins grows, so too will our understanding of how oxygen and hydroxylases dictate signaling events in response to hypoxia.

## 2. Oxygen-Sensing Proteins: Functions and Consequences in the Heart

The oxygen concentration, both global and cellular, has a profound impact on the development, homeostasis, contractility, and injury response of the heart, and the effects can be beneficial or detrimental. Oxygen contributes to the formation of NO, which mediates cardiac and vascular contractility [35] through the oxygen concentration-dependent* S*-nitrosylation of cysteine residues, which in turn mediate calcium flux and excitation-contraction coupling [36]. Oxygen is also the central component in ROS generation. While regarded as molecules with largely negative consequences, ROS can also mediate positive outcomes in regard to cell signaling events. For example, ROS are required for transforming growth factor beta- (TGF*β*-) mediated myofibroblast differentiation in human cells [37], angiotensin II- (ANGII-) mediated bovine and hamster vascular smooth muscle cell proliferation [38], endothelin-mediated cardiac c-fos expression in rat myocytes [39], and other beneficial signaling events [40]. However, ROS are still canonically regarded as harmful molecules, especially from the cardiac-centric perspective, which promotes cell death through mitochondrial damage in rat myocytes [41], lipid peroxidation in rat myocytes [42], chromatin remodeling [43], and protein interactions [44]. In addition to broad antioxidant competition from superoxide dismutases [45–47], ROS are also negatively regulated by other signaling pathways, including the ubiquitin-proteasome system [48], further demonstrating the diverse role that these molecules can play.

Oxygen also serves as a molecule in posttranslational modification. Oxidases are one family of oxygen-conferring enzymes and are critical components with regard to oxygen utilization within the cell. One notable oxidase is cytochrome P450 oxidase, which is involved in myriad hydroxylation reactions [49–51]. NADPH oxidase regulates phagocytosis in neutrophils by generating O_2_^−^, which in turn produces the oxidants H_2_O_2_ and hypochlorous acid, which itself is generated by myeloperoxidase (MPO) [52]. The increase of NADPH oxidase activity also contributes to the progression of atherosclerosis since its superoxide generation leads to the oxidation of low density lipoprotein that exacerbates endothelial dysfunction and foam cell formation [52]. Similarly, FADH_2_ is oxidized by flavoprotein dehydrogenase in the peroxisome during fatty acid oxidation to produce H_2_O_2_, another ROS molecule long known to be central in cardiac ischemia/reperfusion injury [53]. Interestingly, MPO is another key mediator of pathological cardiac events, including atherosclerosis, myocardial injury, and vascular remodeling in both humans and animal models [54–56], most notably through its role of increasing the oxidative potential of its cosubstrate hydrogen peroxide [57]. Elevated levels of MPO in humans correspond to increased risk of cardiovascular disease [58, 59], while human MPO deficiency is cardioprotective [60, 61].

Oxygenases, the other group of enzyme utilizing oxygen as the substrate, can be further divided into two groups depending on whether they catalyze one atom (monooxygenases) or two oxygen atoms (dioxygenases) of oxygen onto the substrate. Monooxygenases are widely distributed in the cell and use coenzymes that use NADPH or FADH_2_ to reduce the second oxygen atom from molecular oxygen to water. Dioxygenases catalyze the oxidation of a substrate without the reduction of one oxygen atom from dioxygen into a water molecule, often by using iron as a cofactor in the reaction. This reaction is accomplished either by incorporating both atoms of molecular oxygen into the substrate or catalyzing molecular oxygen onto multiple substrates [62]. The dioxygenases can further be categorized into three separate groups: iron-dependent enzymes, cambialistic oxygenases, and cofactor independent dioxygenases [63–65], which in turn can be further divided into various superfamilies and families of enzymes whose functions are just now becoming understood.

Of the dioxygenases, two are well known to be factors in cardiovascular disease. The first is cyclooxygenase (COX), which is responsible for the formation of prostanoids, which are oxygenated signaling molecules derived from arachidonic acid and polyunsaturated long-chain fatty acids [66]. These prostanoids, which include prostaglandin and thromboxanes, are involved in the proinflammatory response [67]. The widely popular nonsteroidal anti-inflammatory drugs (NSAIDs) target COX activity but also have highly publicized cardiovascular effects in humans [68]. COX inhibition leads to atherosclerotic plaque destabilization in human patients with recent ischemic events [69] and endothelial dysfunction during hypertension in humans [70] and promotes atherosclerotic lesion formation in LDL-deficient mice [71]. This COX inhibition is thought to induce cardiovascular event by increasing the relative thromboxane levels and decreasing prostacyclin levels in damaged heart tissue [72].

Another well-known subgroup of the dioxygenases is the 2-oxoglutarate (OG)/Fe(II)-dependent superfamily. Also known as *α*-ketoglutarate, 2-OG is an important intermediate and rate-limiting factor of the mitochondrial citric acid cycle (also known as the Krebs or tricarboxylic acid cycle). In the context of oxygenases, most 2-OG-dependent enzymes catalyze the incorporation of one oxygen atom from molecular oxygen into their alcohol product and one into the succinate coproduct [73]. These versatile enzymes are nonheme in character [74], have a double stranded *β*-helix (also referred to as a “jelly-roll”) motif [75], an Fe(II) center that is coordinated by a conserved HX(D/E)X_N_H motif [76], and utilize ascorbate for maximal activity [77], though more recent studies in humans show that ascorbate had little effect on HIF-mediated arterial pressure response to hypoxia [78], making the precise role of ascorbate in HIF-mediated hydroxylation more unclear. Upon substrate binding, 2-OG/Fe(II)-dependent enzymes most commonly catalyze protein hydroxylation [79–81] but are also employed in H3K36 demethylation [82]. This broad class of enzymes is involved in wide-ranging functions like DNA repair [83], metabolism [84], stress response [85], growth factor signaling [86], and not surprisingly, hypoxic response signaling [87, 88].

## 3. Hydroxylases and Hydroxylation in the Heart

One of the best characterized proteins that are modified by 2-OG dependent hydroxylation is HIF. HIF is a heterodimer consisting of a labile *α*-subunit and a stable *β*-subunit [89]. In normoxic conditions, HIF-1*α* protein expression is typically very short lived due to rapid degradation mediated by the VHL E3 ligase [18–20]. This oxygen-dependent degradation of HIF-1*α* is mediated by PHD (also known as EGLN) proteins [88, 90].

There are two proline residues modified in HIF-1*α*, corresponding to P402 (modified by PHD1 and PHD2) and P564 (modified by all three PHDs [PHD1/2/3]) [88]. Through the use of siRNA knockdown experiments in mice, PHD2 has been shown to regulate overall HIF-1*α* stability [91]; however, the precise function and modifications to each proline residue seem to be more complex. Chan et al. [92] demonstrated that residue P564 was hydroxylated prior to residue P402 and that mutation of P564 significantly reduced P402 hydroxylation, but P402 mutation had little effect on P564 hydroxylation state. Additionally, they found that P402 hydroxylation was much more sensitive to physiologic oxygen concentrations relative to P564 hydroxylation [92]. These data indicate that PHDs influence the signaling of HIF under a variety of conditions, demonstrating the divergence and overall complexity of hydroxyl modifications.

HIF activity impacts an array of cardiac phenotypes. Many of the downstream events that occur due to HIF protein stabilization and accumulation (through decreased PHD-mediated hydroxylation) are cardioprotective and are thought to serve as adaptive ischemic preconditioning (IPC) to hypoxia. The IPC phenomenon is thought to protect the heart against a subsequent myocardial damage through the exposure to brief episodes of nonlethal myocardial ischemia and reperfusion [93]. This period of protective preconditioning in mice is initially mediated by HIF (and its corresponding hydroxylation state), possibly through ROS production, or another, as yet determined, transcriptionally independent mechanism [94]. A later ICP effect (sometimes referred to as “SWOP” or Second window of protection) is also seen 24–74 hours after the initiation of preconditioning event [95] and is achieved through HIF's transcriptional induction of the cardioprotective molecules nitric oxide synthase, heme oxygenase 1, and erythropoietin [96]. Further, long-term hypoxia in humans has been shown to significantly reduce mortality due to ischemic heart disease, though the role of HIF in this population was not addressed in this report [97]. Conversely, chronic HIF accumulation has also been shown to be detrimental to cardiac physiology by impairing cardiac metabolism and calcium handling, which are thought to promote cardiac decompensation and premature heart failure in aging mice [98]. Interestingly, PHD proteins in cardiomyocytes are known to interact with the calcium/calmodulin-dependent protein kinase kinase (CaMKK) in rat cardiomyocytes, demonstrating the role of PHDs in cardiac calcium signaling pathways [99]. Further PHD hydroxylases also regulate metabolic activity during stress. Specifically, glucose transporter 1 increases in response to PHD activation, leading to increase in glucose handling [100]. In another report, PHD activation increased glycogen and ATP levels in metabolically stressed mouse cardiomyocytes [101]. Adding yet another layer of complexity, HIF-1*α* has been recently shown to impact cardiac conduction in zebrafish. In* Breakdance* mutant fish, it is hypothesized that the accumulation of HIF-1*α* may partially explain the unexpected longevity and continued development of these fish, even with their characteristic cardiac arrhythmia phenotype [102]. Likewise, PHDs are known to mediate *β*_2_-AR signaling in mice [32], which is responsible for cardiac arrhythmias [103]. While direct evidence is scant, these similarities in HIF- and PHD-mediated cardiac homeostasis may be due to their respective hydroxylation state and enzymatic activity.

In addition to PHD-mediated hydroxylation, HIF-1*α* is also hydroxylated by factor inhibiting HIF (FIH) on asparagine 803 in humans [21]. This hydroxylation event on the transactivation domain of HIF-1*α* prevents its translocation and transcriptional activity by inhibiting p300/CREB-binding protein coactivators binding to HIF-1*α* [21]. Knockdown of FIH in mice has been shown to improve angiogenesis and stem cell mobility post-MI, though this was in addition to simultaneous PHD knockdown [107]. Similarly, FIH has been shown to be an antiangiogenic factor through its association with the E3 ligase Mindbomb in zebrafish [108]. Adding to the complexity of FIH-mediated hydroxylation is its ability to hydroxylate members of the ankyrin repeat family of proteins including the NF-*κ*B inhibitor I*κ*B [104], the receptor protein Notch [105], and the vascular E3 ligase ankyrin repeat and SOCS box containing 6 4 (ASB4) [106], which appears to regulate the angiogenic properties of these proteins in mouse models, possibly in conjunction with NF-*κ*B [109, 110].

The field of cardiac-specific hydroxylation is still emerging. Contributing to this gap is our poor understanding of the hydroxylation event itself. That is, little is known about the role of endogenous hydroxlylase inhibitors, hydroxylases and their target signaling in hypoxia versus normoxia, or putative hydroxylation-site motifs. For example, while many other enzymatic reactions (ubiquitination, acetylation, methylation, etc.) have “counteracting” enzymes that reverse the specific modification (deubiquitinases [111], deacetylases [112], and demethylases [82], resp.), no specific dehydroxylase has been uncovered yet, though speculation of its/their existence has been postulated [113]. Though only just now emerging, there is evidence for the role of HIF-1*α* in nonhypoxic conditions; that is, ANGII, thrombin, various interleukins, cyclicAMP, and other hormones stimulate the accumulation of HIF-1*α* in normoxia (reviewed in [114]). Additionally, glucose is known to stimulate HIF-1*α*-mediated signal transduction in a diabetic mouse model [115]. In addition, HIF-1*α* accumulates in mouse cardiomyocytes during normoxia in response to miR-199a knockdown, increasing HIF-mediated IPC [116]. Though not directly indicative of cardiovascular disease, these pathways and models may yield potential insights into the role of HIF-1*α* in its unhydroxylated state. Another scenario of divergent signaling based on hydroxylation state is with the E3 ligase ASB4. Specifically, ASB4 has been shown to be hydroxylated by FIH [106] and is expressed in the adult heart and brain [106, 117] but is also expressed in hypoxic milieus and functions in placental development and vasculogenesis [106, 118]. This may partially explain instances of a single hydroxylation substrate whose functional role is mediated by the hydroxylation state, independent of other factors such as oxygen availability. Certainly further investigation is warranted into the putative phenomenon of reversible hydroxylation.

Another aspect of hydroxylation that has yet to be fully elucidated is the occurrence and requirement of a hydroxyl-binding site motif. Recent advances in bioinformatics has yielded insights into the amino acid sequence motifs for other enzymatic reactions [119–121], but, while hydroxyl-site motifs have been proposed [122], it remains to be rigorously and empirically determined whether this site (or sites) is required for hydroxylase binding or is a function of happenstance, as other PHD-modified proteins do not contain this sequence [123]. Similarly, unlike ubiquitination, which can seemingly occur on any lysine residue within a protein (though often times with vastly different outcomes) [124], prolyl hydroxylation only occurs on specific proline residues, without any sort of predictability. With the advent of the “-omics” revolution, it stands to reason that hydroxyl modifications are an apropos candidate for high throughput amino acid sequence screen now that better detection methods are available [125] for expanding the discovery of both hydroxylase enzymes and hydroxylated substrates.

## 4. Discussion

The current works demonstrating oxygen-mediated cardiac events highlight the fact that, while we know that these enzymatic reactions are critical for cardiac homeostasis and disease, we are just beginning to realize the impact and scope of such events. The first hydroxylase was discovered in the 1940s [126], but we are only beginning to see the far reaching effect of these enzymes. While the list of substrates is small at present compared to other posttranslationally modifying enzymes, this literature is expanding. From this, we glean the fact that these enzymes are not only important from a basic research standpoint but from a therapeutic and clinical perspective.

Clinically, hydroxylases and hydroxylated proteins such as HIF present a challenging but uniquely rewarding target for drug therapies; that is, while other enzymes require the availability of their modification molecule (ubiquitin for ubiquitination, phosphate for phosphorylation, etc.), the rate limiting factor for hydroxylation is typically the oxygen itself. Therefore, small molecule inhibition/activation, ligand agonism/antagonism, and lipid permeabilization would not be required for changes in hydroxylase efficacy. To increase hydroxylase activity, simple exogenous introduction of recombinant protein would suffice. If accumulating HIF for IPC was desired, via PHD repression, then there is a wide assortment of effective, available treatments. These include cobalt chloride, which can protect the heart in mice by reducing infarct size [127], desferroxamine, an iron chelator that scavenges free radicals and inhibits PHD hydroxylation in rat cardiomyocytes [128], and dimethyloxalylglycine, a broad hydroxylase inhibitor, which also reduced infarct size in rat, mouse, and rabbit animal models due to HIF stabilization [129–131]. HIF itself can be directly targeted as well as a therapeutic intervention; that is, several investigations demonstrate that HIF polymorphisms directly contribute to the pathology of the coronary arteries [132, 133] in human patients. However, HIF has been successfully reintroduced as an adenoviral vector into patients with advanced coronary artery disease as part of a small phase 1 safety study [134].

Over the past decade or so, there have been a growing number of clinical trials of human disease that have focused on PHD proteins and HIF. Small molecule PHD inhibitors have been investigated in their ability to activate HIF and its downstream target genes. Fibrogen, a biotechnology company in California, has designed and implemented several PHD-specific small molecule inhibitors. One compound, FG-2216, has been shown to increase EPO levels [135] in patients with end-stage renal disease, while another compound from the company, FG-4592, has been shown to correct anemia in patients with peritoneal dialysis [136] and patients undergoing dialysis without iron supplementation [137]. While none of these studies are cardiac in nature, these trials serve as a useful proof of principal that these compounds are safe and effective in stabilizing HIF in human patients.

In summary, the oxygen-sensing proteins are an important addition to the complete cardiovascular landscape. Furthering our understanding of the enzymes, substrates, and cofactors involved with oxygen-mediated reactions will not only provide additional knowledge of how heart tissue functions in different oxygen concentrations but also may yield therapeutic interventions for protecting the heart against ischemic injury.

## Figures and Tables

**Table 1:** List of enzymes, functions, and related chemicals.

Name	Function
Ascorbate	Also known as vitamin C, typically functions as an enhancer of 2-OG dioxygenases, though its role in vivo is complex

Cobalt chloride	A chemical inducer of hypoxia-like responses in vivo

Desferroxamine	An iron chelator that inhibits PHD activity

Dimethyloxalylglycine	Broad inhibitor of PHD activity

Dioxygenase	Enzyme that catalyzes two oxygen atoms onto a substrate without the reduction of one oxygen atom from dioxygen into a water molecule, often by using iron as a cofactor in the reaction

Hydroxylase	Enzyme that confers a hydroxyl group (–OH) onto a substrate organic compound

Monooxygenase	Enzyme that catalyzes one oxygen atom onto a substrate, using coenzymes that use NADPH or FADH_2_ to reduce the second oxygen atom from molecular oxygen to water

Oxidase	Enzyme that typically uses a metal or flavin coenzyme to catalyze the oxidation of a substrate without incorporating oxygen into the main product, instead using oxygen as the electron acceptor

Oxygenase	Enzyme that incorporates oxygen molecules into the substrate

Prostaglandins	Lipid compounds that are catalyzed by COX from fatty acids and arachidonic acid

Prostanoids	A class of hormone-like signaling molecules derived from fatty acids, including the prostaglandins and thromboxanes

Superoxide dismutase	Enzyme that protects cells against harmful effects caused by superoxide anion and other free radical ROS

Thromboxanes	Lipid compounds that are catalyzed by COX from fatty acids and arachidonic acid

**Table 2:** List of abbreviations and functions.

Abbreviation	Name	Function
2-OG	2-oxoglutarate	An oxo dicarboxylate obtained by deprotonation of both carboxy groups of 2-oxoglutaric acid

ANGII	Angiotensin II	A hormone that causes vasoconstriction and a subsequent increase in blood pressure

ASB4	Ankyrin repeat and SOCS box containing 4	An E3 ligase hydroxylated by FIH

CaMKK	Calcium/calmodulin-dependent protein kinase kinase	A protein plays a role in the calcium/calmodulin-dependent (CaM) kinase cascade by phosphorylating the downstream kinases CaMK1 and CaMK4

COX	Cyclooxygenase	An enzyme responsible for the formation of prostanoids

cyclicAMP	Cyclic adenosine monophosphate	A derivative of ATP that acts as signaling molecule in many biologic processes

EPO	Erythropoietin	A downstream gene transcribed by HIF, which increases erythropoiesis

FADH	Flavin adenine dinucleotide	A redox cofactor involved in several important metabolic reactions

FIH	Factor inhibiting HIF	A key regulator mediating the cellular response to hypoxia through HIF inhibition

H_2_O_2_	Hydrogen peroxide	A product of superoxide formation involved in damaging cellular effects

HIF	Hypoxia inducible factor	Transcription factors that respond to decreasing oxygen concentrations

IKK*β*	Inhibitor of *κ*B kinase *β*	A protein that leads to the dissociation of the inhibitor/NF-*κ*B complex and ultimately the degradation of the inhibitor

IPC	Ischemic preconditioning	The protection conferred to ischemic myocardium by preceding brief periods of sublethal ischemia

I*κ*B	Inhibitor of kappa B	Inhibits the signaling of NF-*κ*B by masking its nuclear localization signal

LDL	Low density lipoprotein	A class of lipoproteins that confer fat to arterial wall and increase risk of cardiovascular disease

MPO	Myeloperoxidase	A lysosomal protein that converts hypochlorous acid from hydrogen peroxide

NF-*κ*B	Nuclear factor kappa-light-chain-enhancer of activated B cells	A transcription factor that is involved in the immune and inflammatory response

NO	Nitric oxide	A free radical intermediary molecule involved in ischemia

NSAIDs	Nonsteroidal anti-inflammatory drugs	A group of drugs that inhibit COX activity for analgesic and anti-inflammatory effects

O_2_^−^	Superoxide anion	A highly reactive ROS molecule

PHD	Prolyl hydroxylase domain	A group of proteins that hydroxylate proline residues on substrate proteins

PKM2	Pyruvate kinase M2	A coactivator of HIF that is itself hydroxylated by PHD3

ROS	Reactive oxygen species	A class of reactive molecules generated by metabolism that have wide ranging biological functions

SWOP	Second window of protection	A form of delayed ischemic preconditioning that happens 24 hours after initial IPC

TGF*β*	Transforming growth factor beta	A secreted protein involved in a wide array of biological functions, notably in cell differentiation and proliferation

VHL	von Hippel-Lindau	A tumor suppressor E3 ligase that targets HIF for proteasomal degradation

*β*-AR	*β*-adrenergic receptor	A group of G-protein coupled receptors that mediate cAMP concentrations and cardiomyocyte contractility
